# High mortality due to congenital malformations in children aged < 1 year in French Guiana

**DOI:** 10.1186/s12887-018-1372-8

**Published:** 2018-12-22

**Authors:** Mathieu Nacher, Véronique Lambert, Anne Favre, Gabriel Carles, Narcisse Elenga

**Affiliations:** 1 CIC INSERM 1424, Centre Hospitalier Andrée Rosemon, Cayenne, French Guiana; 2 Centre hospitalier de l’Ouest Guyanais, Saint Laurent du Maroni, French Guiana; 3 Service de néonatalogie, Centre Hospitalier Andrée Rosemon, Cayenne, French Guiana; 4 Service de Gynécologie Obstétrique, Centre hospitalier de l’Ouest Guyanais, Saint Laurent du Maroni, French Guiana; 5 Service de pédiatrie, Centre Hospitalier Andrée Rosemon, Cayenne, French Guiana

**Keywords:** Malformations, Circulatory apparatus, Neurological, Mortality, French Guiana

## Abstract

**Background:**

In French Guiana, pregnant women may be exposed to infectious, environmental, and social risks leading to congenital malformation. The objective of the study was to study mortality rates from congenital malformations among infants < 1 year and to compare them with those in mainland France.

**Methods:**

We used the CEPI DC (INSERM) database, which compiles annual data from death certificates in all French territories using the International Classification of Diseases. Annual deaths for French Guiana and mainland France between 2005 and 2015 were compiled. The age category studied was children less than 1 year and deaths from congenital malformations, deformations and chromosomal abnormalities were compiled. Crude risk ratios and 95% confidence intervals were calculated to quantify the excess risk of disease in French Guiana.

**Results:**

In French Guiana between 2005 and 2015 there were 666 deaths of children aged < 1 year, among which, 132 (19.8%) were due to congenital malformations and chromosomal anomalies. Overall the risk ratio of death from congenital malformations and chromosomal anomalies between French Guiana and mainland France was 2.7 (1.5–4.7), *P* < 0.001 for neurological congenital malformations it was 4.8 (1.2–19.7), *P* = 0.01 and for congenital malformations of the circulatory system it was 3.3 (1.5–6.9), *P* = 0.001.

**Conclusions:**

The incidence of death from congenital malformations or chromosomal anomalies in French Guiana was significantly higher than in mainland France. Explanations for this may be infections, genetic causes, nutritional causes, and toxic causes that are prevalent. There is a need to identify factors that predispose children born in French Guiana to having a higher risk of congenital malformations and chromosomal anomalies.

## Introduction

Congenital heart defects (CHD) account for nearly one third of babies with major congenital anomalies diagnosed in Europe [1, 2]. In France the estimated prevalence was estimated at 8.30 (8.00–8.61) per 1000 births [2]. In the USA, congenital heart defects (CHDs) are diagnosed in ‘10 in 1000 to 1500 newborns [3]. In Barbados, congenital malformations of the circulatory system are estimated to be 1.2 per 1000 births (1–1.5) [4]. In Latin America, there are heterogeneities in congenital heart defects ranging from 0.037 to 2.7 per 1000 births [5]. There has been a substantial increase in the prevalence of congenital heart disease worldwide [6]. Proven risk exposures are maternal pre-gestational diabetes mellitus, phenylketonuria, febrile illness, infections, various therapeutic drug exposures, vitamin A use, marijuana use, and exposure to organic solvents. In recent decades progress has led to a decrease in infant mortality and an increase in children with CHD, which raises increasing challenges to care for these patients. In the USA, mortality thus declined from 92.16 per 100,000 population of that age in 1979–1981 and 56.46 per 100,000 population of that age in 1995–1997 [7].

French Guiana is a French overseas territory located on the Guiana shield. Although it has the highest GDP per capita in Latin America, there is a structural lag with mainland France and marked health inequalities within French Guiana. This is often linked with poor pregnancy follow up and preterm delivery [8]. There is a great demographic mix with 2/3rds of the population born outside of French Guiana, a third of the population being foreign born, Creole populations from French Guiana and the Antilles, French Europeans, Maroon populations (descendents of runaway slaves) and indigenous Amerindians. There is widespread admixture, with some populations remaining much more endogamous [9]. This is potentially linked with a greater risk of congenital anomalies [10]. Recently, we described a very high incidence of Pompe’s disease among the Maroon population of French Guiana attributed to a combination of founder effect and endogamy, [11] circumstances that may affect the incidence of other genetic anomalies.

French Guiana has the highest fertility rate in Latin America (3.45) and has an enduring preterm birth problem (13%) and higher rates of infant mortality (9.3 per 1000) than in mainland France(3.5 per 1000) [8, 12]. Despite a free follow-up of all pregnancies with systematic folate supplementation, actual pregnancy follow-up is late and incomplete for a third of women, notably for the most socially vulnerable women [8]. In addition to the recent results on Pompe’s disease, and to recent reports of lead poisoning in pregnant women in French Guiana [13], the Zika virus epidemic focused the attention on pregnancy outcomes of women in French Guiana [14]. In this context, the objective of the present study was to study mortality rates from congenital malformations among infants < 1 year and to compare them with those in mainland France using the same data source to minimize bias.

## Methods

We used the CEPI DC (INSERM) database, which compiles annual data from standardized death certificates in all French territories using the 10th International Classification of Diseases [15]. The death certificates are filled in by a physician who must fill the initial cause of death and the associated causes having contributed to death. Annual deaths for French Guiana and mainland France between 2005 and 2015 were obtained from http://www.cepidc.inserm.fr, which gives the statistics regarding the underlying cause of death. The age category studied was children less than 1 year, which represents the infant mortality from congenital malformations and chromosomal anomalies. The number of deaths attributed to Q20-Q28, or Q00-Q07, or Q00-Q99 was divided by the average number of births over the period. This allowed making comparisons between mainland France and French Guiana. Crude risk ratios and 95% confidence intervals were calculated using Stata’s CSI command in order to quantify the excess risk of disease in French Guiana. The spatial distribution of deaths may have allowed generating hypotheses, but in French Guiana, it was not possible to obtain data about the residence of the children which could have led to inferences regarding the ethnicity. Given the small size of some villages, such data would have made it possible to identify the child, which is forbidden by the Commission Nationale Informatique et Libertés. The data used did not allow us to determine whether there was an autopsy or a dysmorphic evaluation. The analysis uses aggregated data which is publicly available and thus does not require ethical clearance. The analysis was mostly descriptive. Risk ratios were also used to search for a sex-related difference. In order to look for a trend the data was plotted and the Cocchran-Armitage test was used using Royston’s ptrend package for STATA (STATA Corp, College Station, Texas).

## Results

In French Guiana between 2005 and 2015 there were 666 deaths of children aged < 1 year, among which, 132 (19.8%) were due to congenital malformations and chromosomal anomalies. Among these congenital malformations and chromosomal anomalies 71 (10.6%) were attributed to “congenital malformations of the circulatory apparatus”, 17 (2.5%) were neurological congenital malformations, and 44 (6.6%) were other malformations and chromosomal anomalies. In mainland France, in the same time frame there were 29,614 deaths of children aged < 1 year, among which 6200 (20.9%) were attributed to congenital malformations and chromosomal anomalies. Among these 2583 (8.7%) were attributed to “congenital malformations of the circulatory apparatus”, 663 (2.2%) were caused by neurological congenital malformations and 2954 (9.8%) by other malformations and chromosomal anomalies.

When factoring in the number of births, in French Guiana the annual incidence rate of death from “congenital malformations and chromosomal anomalies” was higher than in Mainland France (Table 1). The annual incidence rates of death from “congenital malformations of the circulatory apparatus”, from neurological congenital malformations, and other congenital malformations or chromosomal anomalies were significantly higher than in mainland France.

**Table 1:** Death rates for different congenital or chromosomal anomalies: comparison between French Guiana and France, 2005–2015

	Death rate in children under 1 year of age per 1000 births	Risk ratio French Guiana/France < 1 year
Congenital malformations and chromosomal anomalies (overall)		
French Guiana	2.17	2.6 (2.2–3.1), *P* < 0.0001
Mainland France	0.69	
Neurological congenital malformations		
French Guiana	0.28	3.17 (1.9–5.1), *P* < 0.0001
Mainland France	0.07	
Congenital malformations of the circulatory apparatus		
French Guiana	1.17	3.4 (2.7–4.3), *P* < 0.0001
Mainland France	0.29	
Other congenital malformations or chromosomal anomalies		
French Guiana	0.72	1.8 (1.3–2.5), *P* < 0.0001
Mainland France	0.33	

When comparing male and female deaths there was no statistically significant difference for “congenital malformations of the circulatory apparatus” (*p* = 0.24), neurological congenital malformations (*p* = 0.13), other malformations and chromosomal anomalies (*p* = 0.25). When comparing risk ratios for deaths by gender there was no significant difference (risk ratio = 0.95 (95% CI = 0.6–1.5), risk ratio = 0.8 (95% CI = 0.3–2), and risk ratio = 1. 3 (95% CI = 0.7–2.3), for cardiac, neurological, and other malformations, respectively.

**Fig. 1:**
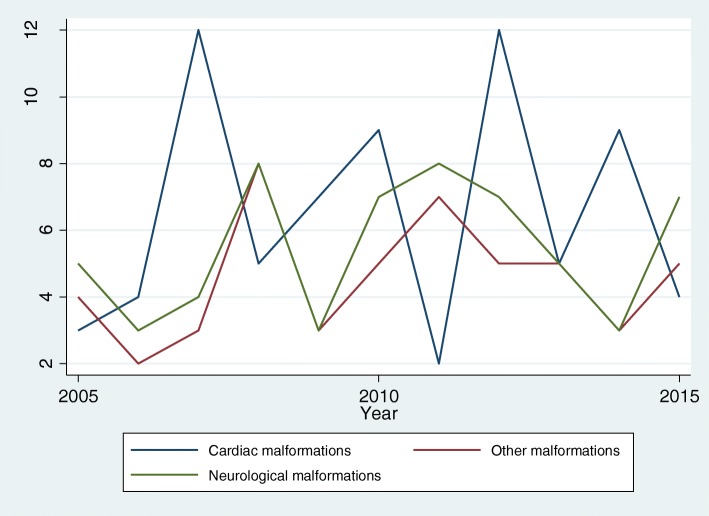
Temporal evolution of the number of deaths of children aged < 1 year in French Guiana caused by congenital malformations

Figure 1 plots the temporal evolution of deaths for congenital malformations of the circulatory apparatus, neurological congenital malformations, and other malformations and chromosomal anomalies. In 2005 there were 3 deaths from circulatory malformations, 5 from neurological malformations, and 4 from other malformations, the number of deaths from circulatory malformations peaked twice at 12 per year, for neurological malformations the highest number of deaths was 8 per year and for other malformations the highest number was 8 per year. In 2015, there were 4 deaths of children aged < 1 year from circulatory malformation, 7 from neurological malformations and 5 from other malformations or chromosomal anomalies. There was no significant temporal trend for the number of deaths from cardiac malformations (*P* = 0.18), neurological malformations (*p* = 0.15), or other malformations (*P* = 0.19).

## Discussion

The main finding of the present study was is that the incidence of congenital malformations of the circulatory apparatus and of neurological congenital malformations are significantly higher than what is observed in Mainland France with the same data source. The high incidence of mortality due to “congenital malformations of the circulatory apparatus” in French Guiana among children aged< 1 year was over 3 times higher than in mainland France. The frequency of death from congenital neurological malformations was also 4.8 times higher in French Guiana than in mainland France, which also raises questions about maternal exposure to infections, toxins, inadequate nutrition and antenatal care that predisposes their infants to develop these congenital malformations. Folate supplementation has been the norm throughout the study period but follow up of pregnancies is often not optimal, notably in the first trimester [8, 16]. These differences using the same instrument could hypothetically be linked to biases in death certificate filling habits, in a context of fewer physicians in French Guiana relative to France. In this scenario, a bias in the diagnostic accuracy of certificates from French Guiana relative to mainland France would inflate the differences. However, physicians in French Guiana are trained in the same university system as those from France therefore this seems an unlikely explanation. It is also possible that there are fewer pregnancy terminations for fetal anomaly for reasons pertaining for lack of access or for cultural barriers. However, another explanation could be that “congenital malformations of the circulatory apparatus” and other congenital pathologies could really be more frequent in French Guiana than in mainland France. We have shown that 3.25% of all mothers in Western French Guiana were heterozygotes for Pompe disease, thus presumably nearly 5% of Maroon mothers being heterozygotes [11]. If we assume prevalence is equivalent in Maroon men and 1 in 4 children of a couple of heterozygotes would get the disease, then 0.05*0.05*0.25*3000 pregnant Maroon women every year the number would be close to 2 per year, which seems to fall short from the observed numbers. Other explanations such as the very high frequency of lead poisoning in French Guiana [13], the frequency of fetal alcohol syndrome [17] the frequency of preterm delivery [18, 19] could be alternative/complementary hypotheses. We did not find any difference between males and females. There was no significant– temporal trend observed. Given the small population of French Guiana and the small number of events annual fluctuations may require a longer observation period to demonstrate any trend. Perhaps a registry of congenital malformations would help understanding and prevention through the collection of individual and detailed information on malformations in French Guiana.

The present study has some important limitations. The use of aggregate data did not allow getting detailed information about each case and thus only gives a crude estimate that would require additional investigation. We only estimated crude risk ratios and could thus not adjust for potential confounding or search for significant effect modifiers. Finally, the quality of the information in death certificates may be variable, notably in remote areas in the interior where health professionals may not always be available to fill death certificates.

## Conclusions

The present paper showed that in French Guiana the incidence of death from congenital malformations of the cardiovascular and neurological system was significantly higher than in mainland France. There are several potential explanations for this high incidence of deaths from congenital malformations: infections during pregnancy [14, 20, 21], exposure to toxic metals [13, 22, 23], fetal alcohol syndrome, diabetes, nutritional deficiencies, social precariousness, preterm birth [8], and endogamy [9, 11]. The policy implications of this observation are that spontaneous abortions, stillbirths, and congenital malformations, should be the focus of specific individual data collection to better describe their nature. Moreover, in a French territory where infant mortality is much higher than in mainland France [24] and where child development and failure in school are also of great concern [25], longitudinal studies should be funded to disentangle the web of potential determinants and find ways to reduce infant mortality and developmental abnormalities in French Guiana.
